# Cyclin-dependent kinase 4 and 6 (CDK4/6) inhibitors: existing and emerging differences

**DOI:** 10.1093/jncics/pkad045

**Published:** 2023-06-27

**Authors:** Stephen Johnston, Anna Emde, Carlos Barrios, Stefanie Srock, Patrick Neven, Miguel Martin, David Cameron, Wolfgang Janni, Michael Gnant

**Affiliations:** Breast Unit, The Royal Marsden NHS Foundation Trust, London, UK; Eli Lilly, Ra’anana, Israel; Grupo Oncoclínicas, Hospital São Lucas, PUCRS, Latin American Cooperative Oncology Group (LACOG), Porto Alegre, RS, Brazil; Lilly, Bad Homburg, Germany; University Hospitals Leuven, Leuven, Belgium; Instituto de Investigación Sanitaria Gregorio Marañon, CIBERONC, Universidad Complutense, Madrid, Spain; Edinburgh Cancer Centre, Institute of Genetics and Cancer, University of Edinburgh, Edinburgh, UK; Department of Gynecology and Obstetrics, University of Ulm, Ulm, Germany; Comprehensive Cancer Center, Medical University of Vienna, Vienna, Austria

## Abstract

The cyclin-dependent kinase 4 and 6 (CDK4/6) inhibitors palbociclib, ribociclib, and abemaciclib are standard-of-care therapy for hormone receptor-positive advanced or metastatic breast cancer, based on randomized trials showing improved progression-free survival for all 3 drugs and overall survival for ribociclib and abemaciclib. Results in early breast cancer are discordant, with sustained improvement in invasive disease-free survival demonstrated for abemaciclib but not other CDK4/6 inhibitors to date. We review nonclinical studies exploring mechanistic differences between the drugs, the impact of continuous dosing on treatment effect, and translational research into potential resistance mechanisms and prognostic and predictive markers. We focus particularly on how emerging findings may help us understand similarities and differences between the available CDK4/6 inhibitors. Even at late-stage clinical development, there remains much to learn about how agents in this class exert their varying effects.

Cyclin-dependent kinases (CDKs) are important regulators of checkpoints that gate entry and ensure orderly progression through the phases of the cell cycle, exerting their effects through various cyclin and CDK combinations (1). The CDK 4 and 6 (CDK4/6) retinoblastoma (Rb) pathway is dysregulated in approximately 80% of human cancers (2). Cyclin D1 and CDK4 contribute to breast tumorigenesis through their interaction with the cell cycle (3,4) and promote proliferation in endocrine-resistant breast cancer cells (5) and, together with Rb, potentially influence breast cancer pathogenesis and progression (3,4,6).

Three oral CDK4/6 inhibitors (palbociclib, ribociclib, and abemaciclib) are established therapies for hormone receptor-positive advanced or metastatic breast cancer (mBC), based on improved progression-free survival (PFS) and, in some cases, overall survival (OS) when combined with standard endocrine therapy (ET). A fourth agent, dalpiciclib, has demonstrated efficacy in hormone receptor-positive mBC in Chinese phase III trials (7,8) but is not available outside China and therefore is not discussed further in this article. Palbociclib and ribociclib are both given on a 3 weeks-on, 1 week-off schedule every 28 days, whereas abemaciclib is administered twice daily without interruption. To date, only ribociclib has shown a clear OS benefit in the first-line metastatic setting in combination with an aromatase inhibitor (AI), and only abemaciclib has demonstrated sustained efficacy in early breast cancer (eBC), as adjuvant treatment for patients at high risk of relapse (Supplementary Figure 1, available online).

All 3 agents target CDK4/6, but more subtle differences relating to selectivity and affinity to CDK4/6 exist, which influence the safety profile and could potentially have an impact on clinical efficacy. Three hypotheses relating to mechanism of action have been proposed for the differing effects of the CDK4/6 inhibitors in mBC and eBC:

- Continuous (abemaciclib) vs intermittent (palbociclib and ribociclib) dosing (9)
- Differential inhibitory activity on CDK4 and CDK6 (10)
- Differential kinome spectra (10)

In this article, we review preclinical and clinical evidence supporting these hypotheses and their clinical application.

## Preclinical data

**Figure 1.**
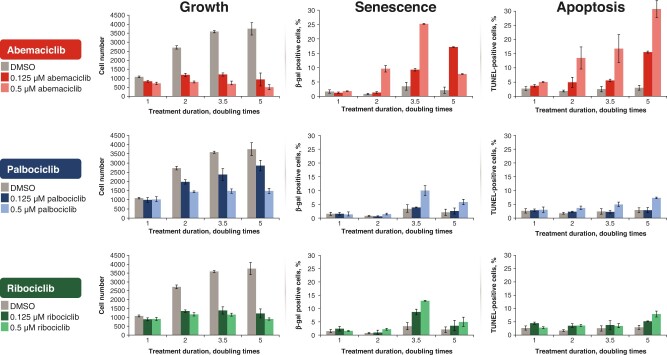
Effect of cyclin-dependent kinase (CDK) 4/6 inhibitors on growth, senescence, and apoptosis. [Reproduced from Torres-Guzman et al. (12).] Data are plotted as the mean (±SD) of 2 experiments for CDK4 and CDK6 inhibitor treatment and of 6 experiments for untreated samples. β-gal  =  β-galactosidase; DMSO = dimethyl sulfoxide; TUNEL = terminal deoxynucleotidyl transferase deoxyuridine triphosphate nick end labeling.

Palbociclib, ribociclib, and abemaciclib inhibit transition from G1 to S phase during the cell cycle via inhibition of CDK4 and CDK6, which they target with varying potency. Palbociclib has similar binding affinity to CDK4/cyclin D3 and CDK6/cyclin D1, whereas ribociclib and abemaciclib have greater binding affinity for CDK4/cyclin D3 [fivefold for ribociclib, ninefold for abemaciclib (10); Supplementary Table 1, available online]. Abemaciclib exhibits the strongest inhibitory capacity for CDK4 and the highest CDK4 to CDK6 inhibition ratio (9,10) (Supplementary Table 1, available online). The lower relative CDK6 inhibition with abemaciclib vs palbociclib and ribociclib may explain the less frequent incidence of myelosuppression, which allows continuous instead of intermittent administration (11). In a preclinical experiment in the hormone receptor-positive Michigan Cancer Foundation-7 (MCF-7) cell line, abemaciclib, palbociclib, and ribociclib showed similar cell growth inhibition, but abemaciclib demonstrated earlier and more pronounced effects on senescence and apoptosis than were observed with palbociclib or ribociclib at equimolar concentrations (12) (Figure 1).

**Figure 2.**
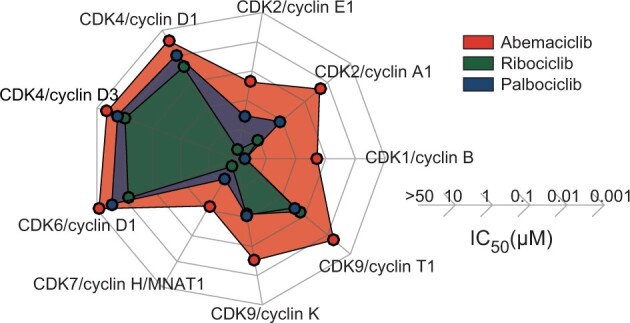
Target spectra of approved cyclin-dependent kinase (CDK) 4/6 inhibitors. [Reproduced from Hafner et al. (13).] IC_50_ values for CDK/cyclin complexes for CDK4/6 inhibitors as measured using purified kinases in vitro. IC_50_ = half maximal inhibitory concentration.

**Figure 3.**
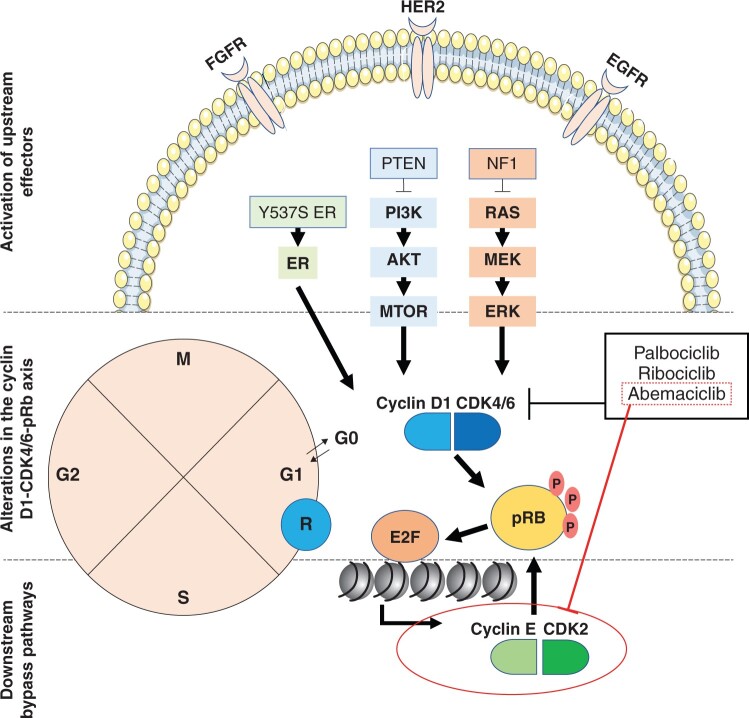
Regulation of the G1–S transition and mechanisms of resistance to cyclin-dependent kinase 4/6 (CDK4/6) inhibitors. [Adapted from Jeselsohn et al. (15).] Fibroblast growth factor receptor (FGFR)-, HER2-, and epidermal growth factor receptor (EGFR)-mediated signals are mediated through phosphoinositide 3-kinase (PI3K) and MAPK and upregulate cyclin D1 directly via estrogen receptor (ER)–mediated signaling, ultimately leading to activation of the cyclin D1-CDK4/6 complex. This complex phosphorylates and de-represses retinoblastoma tumor suppressor protein (pRB), thereby releasing E2F-mediated inhibition and leading to activation of the cyclin E-CDK2 complex, which further phosphorylates pRB. This enables S-phase progression and cell proliferation. Palbociclib, ribociclib, and abemaciclib are selective inhibitors of CDK4/6. Abemaciclib also inhibits CDK2, albeit to a lesser degree. Proposed mechanisms of resistance include activation of upstream effectors (*FGFR* and *ERBB2* [the gene that encodes HER2], *RAS, PTEN*, and *NF1*, Y537S-activating mutation of *ESR1*, which encodes ER); alteration in the cyclin D1-CDK4/6-pRB axis, which includes inactivating mutation of *RB1* (which encodes pRB) and overexpression of CDK6; downstream bypass pathways such as overexpression of cyclin E and activation of the CDK2/cyclin E complex. AKT = protein kinase B; MEK = mitogen-activated protein kinase; MTOR = mammalian target of rapamycin; NF1 = neurofibromatosis type 1.

Abemaciclib inhibits additional kinases more profoundly than other CDK4/6 inhibitors do (13) (Figure 2). Secondary targets include CDK1/cyclin B and CDK2/cyclin A/E complexes (13), which are involved in the transition from S to G2 phase. The clinical impact of inhibiting other kinases remains largely unknown but may contribute to differences between agents, not only in activity and reduced hematologic toxicity with abemaciclib (14) but also in resistance, either primary (intrinsic) or secondary (acquired) (15) (Figure 3).

Resistance to CDK4/6 inhibitors is associated with multiple genomic alterations, including amplification of cyclin E, which promotes CDK2-dependent phosphorylation of retinoblastoma tumor suppressor protein (pRb), amplification of CDK6, and loss of pRb function (16,17). The CDK2/cyclin E pathway represents a potential resistance mechanism for CDK4/6 inhibitors (13,18). CDK4/6 inhibitors inhibit cell proliferation directly by reducing p21 binding to the CDK4/cyclin D1 complex and indirectly through CDK2/cyclin E inhibition via p21 release (19). In predominantly CDK4-dependent cells (such as estrogen receptor-positive [ER+] breast cancer), abemaciclib, ribociclib, and palbociclib displace p21 from the CDK4/cyclin D1 complex (but not CDK6 from CDK6/cyclin D1) (20). The free p21 then binds to the CDK2/cyclin E1 complex, inhibiting CDK2 signaling. Thus, the ratio of CDK4 to CDK6 inhibition may dictate drug sensitivity and resistance (20), and dependency on specific CDKs can be altered by genetic aberrations in cell cycle genes (11). The redundancy and plasticity of the cyclin–CDK network means that either CDK4/6 or CDK2 could drive proliferation in certain circumstances (11).

The greatest efficacy difference between abemaciclib and other CDK4/6 inhibitors was seen in cell lines with a transcriptional profile characterized by higher *CCNE1* (which encodes cyclin E1, implicated in palbociclib resistance), *CDKN1A* (p21, a CDK2/4/6 inhibitor), and *CDKL5* expression and by lower *CDK9* expression. This suggests that preclinical differences between CDK4/6 inhibitors could relate to expression levels of genes targeted to a greater extent by abemaciclib (13).

## Clinical data

### Efficacy in the metastatic setting

Palbociclib, ribociclib, and abemaciclib have shown consistently strong PFS benefit and a trend toward pain improvement (21) in ER+ mBC, supporting administration of CDK4/6 inhibitors in combination with ET at the first available opportunity. However, differences between the available CDK4/6 inhibitors are beginning to emerge.

#### CDK4/6 inhibitors plus AIs as initial therapy

In postmenopausal women receiving AI as initial therapy for ER+ mBC, adding a CDK4/6 inhibitor improved PFS in 3 randomized phase III trials (PALOMA [PALbociclib: Ongoing trials in the MAnagement of breast cancer]-2, MONARCH 3, MONALEESA-2) (22-24). Generally, the trials showed consistent PFS effects across subgroups analyzed. However, in MONARCH 3 (abemaciclib plus AI) (25), there were numerical trends toward greater benefit from abemaciclib (PFS, time to second progression or death, and objective response rate) in patients at higher risk for progression (eg, progesterone receptor negative, high tumor grade, liver metastases, treatment-free interval <3 years) (26,27).

In premenopausal women who had not previously received ET (but may have received 1 line of chemotherapy) for advanced disease, the MONALEESA-7 trial also showed PFS improvements with ribociclib (28).

First-line trials have begun to demonstrate improved OS with the addition of a CDK4/6 inhibitor for ER+ mBC. MONALEESA-7 showed improved OS with the addition of ribociclib to ET in premenopausal women (29,30). MONALEESA-2 (ribociclib plus letrozole) is the only trial that has demonstrated improved OS from a CDK4/6 inhibitor combined with an AI in the first-line postmenopausal setting; median OS exceeded 50 months in both arms (64 months [5.3 years] with ribociclib plus letrozole vs 51 months [4.3 years] with placebo plus letrozole) (31). The OS [and PFS (23)] benefit was particularly pronounced in 227 patients with de novo mBC, which is typically less complex than relapsed eBC and less resistant in the absence of prior adjuvant therapy. In addition, a pooled analysis of 3 MONALEESA trials suggested improved PFS and OS with ribociclib in patients with visceral metastases, including subsets with a worse prognosis, such as liver metastases or at least 3 metastatic sites (32). In contrast, the PALOMA-2 trial showed no difference in OS with the addition of palbociclib to letrozole, and the trend for OS in the subgroup with de novo mBC was in the opposite direction, favoring the placebo arm (33). In the first-line MONARCH 3 trial of abemaciclib, median OS was 67 months (5.6 years) with abemaciclib plus AI and 55 months (4.5 years) with placebo plus AI at the second interim OS analysis (34,35). As the observed hazard ratio (HR) of 0.75 (95% confidence interval [CI] = 0.58 to 0.97) did not reach the prespecified statistical significance boundary, the trial continues to follow OS. In all these trials, median OS exceeded 50 months in both treatment groups. First-line trials may enroll populations with a particularly good prognosis, characterized by endocrine sensitivity, long treatment-free interval, and a high proportion of de novo mBC. Indeed, the percentage of patients with de novo mBC was 34% in MONALEESA-2 (31), 37% in PALOMA-2 (33), and 40% in MONARCH 3 (25).

Of note, few patients in MONALEESA-2 and PALOMA-2 received CDK4/6 inhibitors at progression (33% vs 27%, respectively, in the control arms; 22% vs 12%, respectively, in the CDK4/6 inhibitor arms). Crossover and treatment beyond progression will be important when interpreting mature OS results across all 3 first-line trials because this approach is frequently adopted in everyday practice (36).

All international guidelines recommend ET as the preferred first-line treatment for ER+ mBC, but the preferred option for patients with symptomatic visceral metastases has remained uncertain. However, recently the randomized RIGHT Choice phase II trial showed longer PFS (primary endpoint) with first-line ribociclib plus ET vs a chemotherapy doublet in pre- or perimenopausal patients with hormone receptor-positive HER2-negative (HER2-) advanced breast cancer with aggressive clinical features (rapidly progressing or highly symptomatic disease, including life-threatening visceral crisis) (37). Median PFS was doubled with ribociclib plus ET (24 months vs 12 months), supporting the superiority of ET plus CDK4/6 inhibitor therapy over chemotherapy in this setting.

#### CDK4/6 inhibitors plus fulvestrant after ET

CDK4/6 inhibitors are also standard of care for patients developing progressive metastatic disease during single-agent ET (either tamoxifen or an AI) in the adjuvant or first-line mBC setting (ie, endocrine resistant). In 3 randomized phase III trials (PALOMA-3 with palbociclib; MONALEESA-3 with ribociclib; MONARCH 2 with abemaciclib), combining CDK4/6 inhibitors with fulvestrant as second-line therapy improved PFS compared with fulvestrant alone (38-40). OS was improved with ribociclib and abemaciclib (41,42) but not palbociclib (43). A notable difference between PALOMA-3 and the other trials was the inclusion of patients previously treated with chemotherapy for mBC, representing approximately one-third of the study population.

Although the relevance of these results as second-line therapy may be diminishing given the widespread use of CDK4/6 inhibitors as first-line therapy (and in the future, as adjuvant therapy), they may hint at differences within the class of CDK4/6 inhibitors. In the second-line setting when disease is progressing on a CDK4/6 inhibitor plus an AI, it is unclear whether CDK4/6 inhibition should continue with the same or a different inhibitor and whether there should be a concomitant change in ET partner. Recent results from the randomized phase II MAINTAIN trial indicate that switching CDK4/6 inhibitors (in this case, from palbociclib to ribociclib) and switching ET to fulvestrant after progression almost doubled PFS compared with changing to fulvestrant alone (44). However, in the 3-arm randomized phase II Palbociclib After CDK and Endocrine therapy (PACE) trial in patients whose disease progressed on a CDK4/6 inhibitor (mainly palbociclib) plus AI, the combination of palbociclib and fulvestrant (ie, keeping the same CDK4/6 inhibitor and switching the ET partner) did not improve PFS compared with fulvestrant alone (45).

These data provide little support for staying on the same CDK4/6 inhibitor at disease progression but possibly some evidence to support switching the individual CDK4/6 inhibitor. A slightly different switching approach was explored in the PAlbociclib and circulating tumor DNA for *ESR1* mutation detection (PADA)-1 trial in which patients with rising *ESR1* mutations (a molecular mechanism of resistance to AI therapy) detected in circulating tumor DNA during first-line AI and palbociclib therapy were randomly assigned either to continue the same treatment or to switch to fulvestrant with palbociclib. Patients switching to fulvestrant plus palbociclib after detection of an *ESR1* mutation without clinical disease progression had longer PFS than those who continued on AI and palbociclib (median 11.9 vs 5.7 months, respectively; stratified HR = 0.61, 95% CI = 0.43 to 0.86; *P* = .004) (46). This suggests that biomarker monitoring could become a strategy to detect early molecular progression and allow a switch in ET from an AI to a selective ER degrader (SERD) that targets mutated ER.

#### Resistance related to cyclin E1

In mBC, most patients have received previous treatment in either the adjuvant or the metastatic setting, which may alter tumor biology (47). Consequently, except for first-line therapy of de novo mBC, the focus is usually on acquired resistance (generated through selection pressure under therapy). In PALOMA-3, higher mRNA expression of *CCNE1* (cyclin E1 gene) was associated with relative resistance to palbociclib (48), providing the strongest clinical evidence for relevant biological changes at the clinical level. In MONALEESA-7, premenopausal women with alterations in *CCND1* (cyclin D1 gene) derived enhanced benefit from the addition of ribociclib to ET (49). Translational research studies in CDK4/6 inhibitor trials have sought to determine biomarkers of resistance to therapy (either in biopsies of metastatic disease or in circulating tumor DNA), either detected before starting therapy or subsequently acquired during treatment. To date, there is no biomarker that prevents initial treatment with a CDK4/6 inhibitor. In contrast, detection of acquired biomarkers, such as *ESR1* mutations or *PIK3CA* mutations, will guide the choice of subsequent targeted therapies, and the use of these biomarkers in this setting after CDK4/6 inhibitors in mBC is now recommended as standard practice (50).

### Efficacy in the neoadjuvant setting

**Figure 4.**
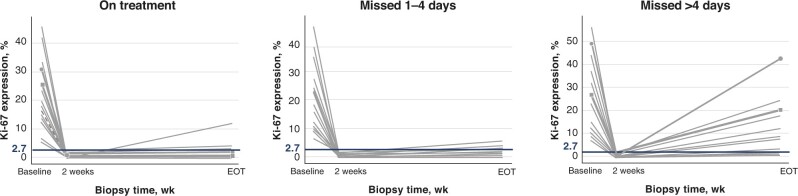
Impact of abemaciclib treatment interruptions on cell proliferation inhibition (exploratory analysis of neoMONARCH). [Adapted from Hurvitz et al. (51).] Plots show Ki-67 expression for individual patients with complete cell cycle arrest at 2 weeks (regardless of treatment) who stayed on treatment (n  =  26), discontinued treatment up to 4 days before biopsy (n  =  19), or discontinued treatment more than 4 days before biopsy (n = 13). Ki-67 values greater than 2.7 considered as Ki-67 rebound. EOT = end of treatment.

Evidence from abemaciclib and palbociclib neoadjuvant trials suggests that continuous dosing is important in inhibiting cell proliferation, as assessed by the proliferation marker Ki-67. In the phase II neoMONARCH trial, abemaciclib (with or without anastrozole) was associated with greater Ki-67 suppression and complete cell cycle arrest (CCCA) than anastrozole alone (51). Exploratory analyses revealed Ki-67 rebound in 69% of patients who discontinued treatment more than 4 days before biopsy compared with only 11% who discontinued treatment 1-4 days before biopsy (Figure 4), suggesting that in the clinical setting, continuous dosing of CDK4/6 inhibitors may be required to maintain cell cycle arrest and proliferation inhibition. Similarly, in the randomized phase II PALLET trial (52), CCCA was observed at week 14 in 90% of patients receiving palbociclib plus letrozole vs 58% receiving letrozole alone. After palbociclib withdrawal, rebound was indicated by rapid Ki-67 increase in a time frame similar to the week off palbociclib in the intermittent schedule (53).

Consistent findings in PALLET and neoMONARCH suggest that tumor suppression may require ongoing drug exposure in the neoadjuvant (and possibly adjuvant) settings. In contrast, in the FELINE randomized phase II neoadjuvant trial, continuous dosing of ribociclib plus letrozole showed no benefit over intermittent dosing (54).

### Efficacy in the adjuvant setting

Four large, randomized phase III trials have been designed to evaluate CDK4/6 inhibitors as adjuvant therapy for eBC, all with a primary endpoint of invasive disease-free survival (IDFS). Of the 3 trials that have formally reported results, only 1 (evaluating abemaciclib) has demonstrated efficacy in eBC.

#### Palbociclib (PALbociclib coLlaborative Adjuvant Study [PALLAS] and PENELOPE-B)

In PALLAS, the first randomized phase III trial of adjuvant CDK4/6 therapy to report, patients with hormone receptor-positive HER2- stage II-III eBC were randomly assigned to adjuvant ET with or without 2 years of adjuvant palbociclib. IDFS was not improved in the investigational arm at the second interim analysis, and palbociclib treatment was discontinued for futility (55). The IDFS HR at the final analysis was 0.96 (95% CI = 0.81 to 1.14) (56). Four-year IDFS rates were 84.2% with palbociclib plus ET vs 84.5% with ET alone.

The second trial to report was the double-blind placebo-controlled randomized phase III PENELOPE-B trial, which enrolled women with high-risk hormone receptor-positive HER2- primary breast cancer with residual invasive disease after taxane-containing neoadjuvant chemotherapy (57). At the final analysis, the addition of 1 year of palbociclib to adjuvant ET failed to demonstrate improved IDFS (HR = 0.93, 95% repeated CI = 0.74 to 1.17).

#### Ribociclib (NATALEE)

The open-label randomized phase III NATALEE trial (NCT03701334) is evaluating the addition of 3 years of ribociclib to 5 years of ET as adjuvant treatment for hormone receptor-positive HER2- stage II (N0 with grade 2 or 3 and/or Ki-67 ≥ 20%, or N1) or stage III eBC (58). Initial diagnosis had to be no more than 18 months before random assignment, and patients had to have completed chemotherapy and radiotherapy (if indicated). It was announced recently that interim results are positive (59).

#### Abemaciclib (monarchE)

In contrast to PALLAS and PENELOPE-B, the monarchE randomized phase III trial demonstrated improved outcomes with the addition of adjuvant abemaciclib to adjuvant ET. The monarchE trial aimed to enroll a population at high risk of recurrence despite best available therapy (95% of patients received chemotherapy either pre- or postsurgery). Cohort 1 enrolled 5120 patients with at least 4 positive axillary lymph nodes (ALNs) or 1-3 positive ALNs together with grade 3 disease and/or tumor size of at least 5 cm. Cohort 2, added following a regulatory request, enrolled 517 patients with 1-3 positive ALNs with lower grade and tumor size (grade <3 and tumor size <5 cm) but biologically high risk based on proliferation rate (Ki-67 ≥ 20%). Patients were randomly assigned 1:1 to receive at least 5 years of adjuvant ET with or without abemaciclib for 2 years (60,61).

A planned interim analysis after 15.5 months of follow-up demonstrated improved IDFS (primary endpoint) with the addition of abemaciclib in the intention-to-treat population (60). An updated analysis after a median of 27 months of follow-up, performed at the request of regulatory authorities, showed an IDFS HR of 0.70 (95% CI = 0.59 to 0.82) (61). Most recently, the prespecified interim OS analysis (median follow-up of 42 months) after all patients had completed or discontinued abemaciclib showed an improved IDFS HR of 0.66 (95% CI = 0.58 to 0.76) with a distant relapse-free survival HR of 0.66 (95% CI = 0.57 to 0.77) (62). The magnitude of benefit from abemaciclib appears to be increasing over time and extends beyond completion of 2 years of abemaciclib treatment. The absolute benefit was 2.6% at 2 years, 4.1% at 3 years, and 5.9% at 4 years (62). This suggests a potential carryover effect of treatment, which has been seen previously with adjuvant ET. OS results are immature with deaths in 5.6% of the abemaciclib group and 6.1% of the placebo group (OS HR = 0.93, 95% CI = 0.75 to 1.15) (62).

Prespecified subgroup analyses revealed no obvious differential effects, although a particularly large benefit was observed in premenopausal patients and those previously treated with neoadjuvant chemotherapy. Importantly, results in the ET-alone control arm were as expected for a high-risk node-positive population: 20% had relapsed at 4 years despite standard-of-care therapy with locoregional treatment, chemotherapy, and ET monotherapy. The benefit from abemaciclib in this high-risk group represents a clinically important advance. Nevertheless, it remains important to follow patients with relapse after stopping abemaciclib. This may inform on the role (or not) of reintroducing abemaciclib (or another CDK4/6 inhibitor) at relapse.

#### Ki-67

In Europe, abemaciclib is approved in combination with ET as adjuvant treatment for patients with hormone receptor-positive HER2- node-positive eBC at high risk of recurrence. Controversially, the initial US Food and Drug Administration (FDA) approval of adjuvant abemaciclib plus ET was restricted to patients with Ki-67 greater than 20%, based on the FDA’s assessment of benefit to risk at 27 months’ follow-up (63-65). Importantly, cohort 2 of monarchE started enrolling 1 year after cohort 1 and represents only 9% of the population, with fewer events and a slightly smaller treatment effect observed in cohort 2. The primary analysis of the trial is based on the intention-to-treat population (cohorts 1 and 2 together), with further analyses prespecified.

Following FDA approval of adjuvant abemaciclib to a restricted subgroup of patients, the National Comprehensive Cancer Network and American Society of Clinical Oncology guidelines were updated, broadening eligibility to match monarchE criteria so as to include all hormone receptor-positive HER2- node-positive high-risk eBC (66,67). This avoids some of the challenges related to reliability and reproducibility of Ki-67 assessment highlighted by the International Ki-67 Working Group, including variability of assessments, differences in antibodies, inconsistencies in pre-analytic handling, and lack of consensus over the optimal cutoff to determine high expression (68,69).

Ki-67 showed a prognostic effect in monarchE (at least for early events in the first 4 years) (Supplementary Figure 2, available online). Patients with high Ki-67 had a worse outcome than those with low Ki-67, irrespective of treatment. However, Ki-67 did not show predictive value, and high Ki-67 does not identify patients deriving greater benefit from abemaciclib, as demonstrated by similar HRs and absolute differences in 4-year IDFS rates within cohort 1 for patients with either low or high Ki-67 (62). With this latest analysis of the monarchE data, the FDA recently extended their approval for adjuvant abemaciclib to all patients eligible for cohort 1, without requiring Ki-67 assessment (70).

Although there is no evidence to suggest that baseline Ki-67 is a predictive marker for abemaciclib efficacy, Ki-67 appears to play a role as a dynamic marker. The West German Study Group Adjuvant Dynamic marker-Adjusted Personalized Therapy (ADAPT)-HR+/HER2- trial indicated that Ki-67 levels after 3 weeks of ET can be used to guide systemic therapy according to risk, thus enabling personalized treatment (71). Patients with low-risk breast cancer, no more than 3 involved lymph nodes, and a response to ET as determined by a Ki-67 score of no more than 10% at 3 weeks could be spared chemotherapy without a detrimental effect on disease-free survival. These findings led to the WGS-ADAPTcycle randomized phase III trial (NCT04055493), which is comparing ribociclib plus ET vs chemotherapy as adjuvant dynamic marker-adjusted (neo)adjuvant treatment for intermediate-risk hormone receptor-positive HER2- eBC. In addition, the WGS-ADAPTlate randomized phase III trial (NCT04565054) is comparing abemaciclib plus standard ET vs standard ET alone as adjuvant dynamic marker-adjusted therapy for high-risk hormone receptor-positive HER2- eBC.

Further evidence for the role of on-treatment Ki-67 changes comes from the Peri-Operative Endocrine Therapy—Individualising Care (POETIC) trial in postmenopausal women with ER+ eBC (72). The effect of peri-operative AI therapy on Ki-67 values could be used to differentiate between women at high biological risk of recurrence because of endocrine resistance vs those who could be adequately treated with standard adjuvant ET. The change from baseline in Ki-67 provided a better prediction of outcome than baseline Ki-67 alone. Building on these findings, the POETIC-A randomized phase III trial (NCT04584853) of adjuvant abemaciclib will explore indicators of resistance using dynamic changes in proliferation to identify endocrine-resistant tumors, moving beyond anatomical risk to look at biological risk.

#### Resistance related to cyclin E1 in eBC

Almost 2 decades ago, Keyomarsi et al. (73) reported the prognostic effects of cyclin E and low molecular weight cyclin E in patients with (predominantly early) breast cancer. High total cyclin E level and high levels of low molecular weight cyclin E1 expression (measured by Western blotting and immunohistochemistry) were associated with worse OS.

A more recent analysis of patients with stage I-III eBC (treatment-naïve all-comer population, irrespective of ER and HER2 status) showed that levels of cyclin E and its co-activator phospho-CDK2 were strongly associated with recurrence-free survival (74). Combining cyclin E with phospho-CDK2 enhanced prognostic power in eBC: patients whose cytoplasmic staining was negative for cyclin E and phospho-CDK2 had better recurrence-free survival than those positive for either or both markers. Of note, patients with the best prognosis (negative for both markers) were more likely to have clinical characteristics associated with a better prognosis (low-grade, ER-positive, progesterone-positive, HER2- tumors). In this analysis, patients did not receive CDK4/6 inhibitors, and prognostic and resistance characteristics cannot be distinguished.

Most recently, gene expression data from the PALLET trial of neoadjuvant palbociclib indicated that higher baseline Ki-67, cleaved-polyADP ribose polymerase (PARP), and *CCNE1* levels were associated with a lower likelihood of CCCA at 14 weeks. *CCNE1* levels decreased by 82% in tumors showing CCCA but were unchanged in those without CCCA (53). Whether *CCNE1* expression is a true biomarker of resistance to CDK4/6 inhibitors in eBC remains unclear. In both PALLAS and monarchE trials, large translational research studies are ongoing to address all potential biomarkers that might ultimately allow us to understand who benefits most or least from these therapies in this setting.

### Safety, patient-reported outcomes, and patient preference

Supplementary Table 2 (available online) summarizes key safety results from phase III trials of the 3 agents across mBC and eBC settings. Hematologic adverse events and gastrointestinal effects are characteristic of all 3 agents, although hematologic effects are more common with palbociclib and ribociclib, whereas diarrhea and abdominal pain are more common with abemaciclib. In phase III trials, infections and alopecia were reported more frequently with palbociclib and ribociclib; liver toxicity was reported more often with ribociclib and, to a lesser extent, abemaciclib.

Overall, the addition of CDK4/6 inhibitors to ET does not appear to worsen patients’ health-related quality of life, and several studies have shown a trend toward pain improvement with CDK4/6 inhibitors (21). Differences between the individual drugs reflect characteristics of their safety profiles. For example, changes in diarrhea were worse in the abemaciclib arm than the control arm in MONARCH 2 (75) and MONARCH 3 (76), although this effect was not seen in monarchE (77) in eBC.

In a preference study conducted in the United States focusing on safety (but not efficacy) attributes influencing treatment choice, the risk of diarrhea and grade 3 or 4 neutropenia were key drivers of treatment preference for both oncologists and patients with mBC and were considered substantially more important than the risk of dose reduction for adverse events (78). Oncologists also considered the need for electrocardiogram monitoring to be important. Dosing frequency was not a key factor in patient and oncologist preferences. In a similar study exploring treatment preference in the adjuvant setting, patients and oncologists ranked 5-year IDFS rate as considerably more important than diarrhea or neutropenia risk (79).

## Discussion

Emerging data from large, randomized trials of approved CDK4/6 inhibitors are deepening our understanding and may begin to explain similarities and differences between individual agents and the divergent results seen in the mBC and eBC settings.

### Continuous dosing

It seems intuitive to expect better efficacy with continuous cell cycle blockade, as seen with many targeted agents. Mechanistically, sustained treatment may be necessary to ensure drug exposure when cells are susceptible to apoptosis and/or to block cells from reproliferating.

The preclinical results described above suggest that continuous suppression of the CDK pathway provides better control of cell proliferation than can be achieved with cyclical peaks and troughs of suppression and nonsuppression. Additional clinical evidence stems from the neoMONARCH and PALLET neoadjuvant clinical trials, which indicate very rapid rebound after CDK4/6 inhibitor treatment withdrawal. However, clinical results for ribociclib in mBC challenge this hypothesis, demonstrating high efficacy despite the intermittent administration schedule. Dosing schedules are typically based on feasibility and toxicity, with treatment breaks or drug holidays incorporated into the schedule if required to mitigate toxicity, but there is little clinical evidence to justify a continuous metronomic schedule over an intermittent schedule for most agents. An alternative approach for managing toxicity is to lower the starting dose, which can allow continuous dosing and thus continuous target blockade. The traditional (chemotherapy) dose-escalation approach to schedule selection may not be appropriate for targeted agents, and for many anticancer drugs, the dose used in clinical practice may be higher than necessary. Dose reductions and omissions are part of the side-effect management with CDK4/6 inhibitors, tailoring treatment to an individually tolerable dose. A growing body of evidence shows that the efficacy of CDK4/6 inhibitors in mBC is not impaired by dose adjustment to manage toxicity (80-82).

Given the findings in mBC, it is difficult to attribute the efficacy of abemaciclib entirely to its continuous dosing schedule. Data in the neoadjuvant setting suggest that continuous dosing is important in eBC, but more clinical evidence is required to understand its relevance in the metastatic setting. For example, are there biomarkers to determine whether cell cycle control with continuous dosing is important in mBC? From a cell kinetics perspective, cells in multiple metastatic sites are either actively in cell cycle or more dormant, and therefore interruptions in treatment are unlikely to result in major changes in disease control. Biologically, it is unclear what impact CDK4/6 inhibitors have on micrometastatic dormant cells (11), and it is difficult to prove the clinical relevance of senescence and apoptosis, despite demonstration of suppression during drug treatment and loss of suppression after treatment withdrawal.

### Differences between early and metastatic disease settings

In mBC, differences between the various CDK4/6 agents are only beginning to emerge (Supplementary Figure 1, available online); however, reevaluation of contrasting results from the 3 randomized phase III trials assessing CDK4/6 inhibitors in eBC (55–57,60–62) suggests potential differences between the drugs. There are essentially 4 hypotheses for varying trial outcomes: differing risk level between study populations, differences in drug exposure, continuous vs intermittent dosing, and true differences between the drugs (83,84). Further analyses refute the first 2 options. monarchE enrolled a high-risk population, whereas PALLAS included a mixed population of patients at intermediate and high risk. However, there was no benefit from palbociclib in patients with N2 or N3 disease in PALLAS (N2 HR = 0.91, 95% CI = 0.66 to 1.25; N3 HR = 0.89, 95% CI = 0.64 to 1.21), although this subgroup analysis had limited power (56). In addition, PENELOPE-B enrolled a high-risk population (no pathologic complete response after taxane-containing neoadjuvant chemotherapy and grade ≥3 or grade 2 and ypN+) (57). Differences in drug exposure do not explain the disparate outcomes. Treatment discontinuation rates were higher in PALLAS (42%) than monarchE (17%), potentially compromising treatment exposure and efficacy. However, post hoc analyses of PALLAS showed no differential effect with higher vs lower palbociclib exposure (85). In the double-blind placebo-controlled PENELOPE-B trial, 20% of patients stopped treatment prematurely (planned treatment duration: 1 year).

This leaves 2 of the 4 possible explanations mentioned above: continuous vs intermittent dosing or true differences between the drugs. Treatment withdrawal effects in the neoadjuvant setting support the need for continuous dosing schedules in eBC. In addition, the differential inhibitory activity on CDK4 may play a role. Actively proliferating disease may act differently from dormant disease, and the difference between treating many thousand cells in the macroscopic setting vs a few cells in the microscopic setting may be important to the activity of CDK4/6 inhibitors (55). Continuous dosing may be more relevant in eBC than mBC (where all CDK4/6 inhibitors show PFS benefit, albeit with differing impacts on OS). However, news of positive results for adjuvant ribociclib in the NATALEE trial (59) challenge the continuous dosing hypothesis.

The final hypothesis is a true difference between drugs. Recurrences during the first 2 years on adjuvant ET correspond to primary endocrine resistance (86). In primary endocrine-resistant mBC, abemaciclib has demonstrated OS benefit, whereas palbociclib has not. Results in eBC (60,62), kinome mapping (10), and a more pronounced effect of abemaciclib in aggressive, highly proliferative mBC (26,27) support differences between the drugs.

### Role of CDK2/cyclin E1 signaling

Emerging evidence suggests CDK2/cyclin E1 signaling is an important resistance mechanism for palbociclib. Overactivation of CDK2 activity may limit the potency of CDK4/6 inhibitors, and individual cancer cells with high cyclin E1 levels may increase CDK2 activity thus bypassing CDK4/6 blockade (18,87). However, without clinical validation or reliable measurement of CDK2/cyclin E1 signaling, it is difficult to translate laboratory-based hypotheses to clinical practice. Several questions remain, including whether the mechanism differentiates between abemaciclib, ribociclib, and palbociclib and whether cyclin E activation can explain differences between the drugs. Translational results support the hypothesis of potential partial cross-resistance between ET and palbociclib (88), which may help explain why palbociclib and abemaciclib show different results in eBC.

The relevance of the findings above to clinical practice pivots on the potential of abemaciclib to overcome resistance resulting from cyclin E amplification by blocking CDK2/cyclin E. Theoretically, abemaciclib could be beneficial in palbociclib-resistant tumors with high cyclin E1 expression (13). The postMonarch trial (NCT05169567) is exploring whether this marker may identify patients with progression on ribociclib or palbociclib who may subsequently respond to abemaciclib.

### Unanswered questions and outlook

The wealth of data in mBC provides robust evidence that CDK4/6 inhibitors represent a powerful companion that magnifies the efficacy and benefit of ET. For patients at high risk of recurrence, there is strong evidence that CDK4/6 inhibition modulates endocrine signaling, transforming ET. Mature OS results (particularly from MONARCH 3) and results from ongoing trials (eg, the HARMONIA [NCT05207709] trial, which is comparing ribociclib vs palbociclib as therapy for HER2-enriched mBC) may help tease out differences between the CDK4/6 inhibitors. However, while searching for differences between the drugs in our attempts to optimize outcomes for patients, we should not overlook the global disparity in access to CDK4/6 inhibitors, which means that in some health-care systems, none of these drugs offering the chance of survival beyond 5 years is available for women with hormone receptor-positive mBC.

Abemaciclib (and potentially ribociclib) bring the same promise to the eBC setting. It remains to be seen how the CDK4/6 landscape will evolve in eBC as translational research unfolds and further trials report. Furthermore, as CDK4/6 inhibitors are incorporated into adjuvant treatment, it is important to elucidate the activity of CDK4/6 inhibitor reexposure at relapse. Mechanisms of resistance are multifactorial and heterogeneous, and therefore clinical approaches are likely to reflect this heterogeneity, relying on molecular characteristics to determine whether further CDK4/6 inhibition is likely to be effective or whether alternative pathways should be targeted (89). Treatment selection is already guided in part by somatic mutation testing after progression on CDK4/6 inhibitors, and molecular characteristics play an important role in treatment selection given the array of targeted strategies now available (90).

One of the most important unanswered questions in eBC is whether tumors other than high-risk tumors, as identified by clinicopathological features and/or Ki-67, benefit from abemaciclib. To expand the abemaciclib indication from high-risk to intermediate-risk eBC, a trial would need to be designed for intermediate-risk patients in whom there is uncertainty whether to administer chemotherapy. This population is difficult to define as it is often represented by discordant prognostic factors (eg, high Ki-67 but low risk according to other criteria or 2 positive ALNs but no other strong risk factors) but represents patients for whom choosing between the risks of overtreatment or undertreatment is challenging. From a clinical academic perspective, these patients may be candidates for CDK4/6 inhibitors instead of chemotherapy, and such a trial should be prioritized. Results from the NATALEE trial may also provide insight into the role of CDK4/6 inhibitors in this setting.

## Supplementary Material

pkad045_Supplementary_Data

## Data Availability

No new data were generated or analyzed in support of this research.
