# Supplementary material for: Cyclin-dependent kinase 4 and 6 (CDK4/6) inhibitors: existing and emerging differences
Source: JNCI Cancer Spectr. 2023 Jun 27;7(4):pkad045. doi: 10.1093/jncics/pkad045 (PMC10415176; doi:10.1093/jncics/pkad045)
Supplement: pkad045_Supplementary_Data [file pkad045_supplementary_data.pdf]

Supplementary Table 1. Independent external analysis comparing three CDK4/6 inhibitors with the same assay. (Adapted from Chen et al. [1])

Analysis (nmol/L)	Abemaciclib	Palbociclib	Ribociclib
Biochemical			
CDK1/cyclinA2 K_i	330 ± 90	>1400	>1400
CDK2/cyclinE1 K_i	150 ± 60	>2500	>2500
CDK4/cyclinD3 K_i	0.07 ± 0.01	0.26 ± 0.03	0.53 ± 0.08
CDK5/p35 K_i	86 ± 12	>2000	>2000
CDK6/cyclinD1 K_i	0.52 ± 0.17	0.26 ± 0.07	2.3 ± 0.3
CDK7/cyclinH/MAT1 K_i	220 ± 10	>2000	>2000
CDK9/cyclinT1 K_i	4.1 ± 1.3	150 ± 10	190 ± 20
Pharmacodynamics in cells			
MCF-7 IC_{50}			
pRb-Ser807	13 ± 4	20 ± 7.5	89 ± 36
pRb-Ser780	6.1 ± 1.4	9.3 ± 1.5	31 ± 8.2
T47D IC_{50}			
pRb-Ser807	10 ± 2.9	21 ± 3.3	73 ± 18
pRb-Ser780	8.9 ± 5.9	17 ± 10	51 ± 30
Cell proliferation			
Breast cancer (MCF-7) IC_{50}	86 ± 14	120 ± 60	200 ± 90
Breast cancer (T47D) IC_{50}	94 ± 41	130 ± 80	260 ± 130

CDK = cyclin-dependent kinases; IC_{50} = half maximal inhibitory concentration; K_i = binding affinity; MCF-7 = Michigan Cancer Foundation-7; pRB = retinoblastoma tumor suppressor protein.

Reference

1. Chen P, Lee NV, Hu W, et al. Spectrum and degree of CDK drug interactions predicts clinical performance. *Mol Cancer Ther*. 2016;15(10):2273–2281.

Supplementary Table 2. Overview of safety profiles and treatment discontinuation rates in phase III trials of CDK4/6 inhibitors in early and advanced breast cancer. AI = aromatase inhibitor; ALT = alanine aminotransferase; AST = aspartate aminotransferase; ET = endocrine therapy; WBC = white blood cell.

Patients, n (%)	Palbociclib	Ribociclib	Abemaciclib
**Advanced breast cancer**			
Most common AEs (all grades)[a]	**PALOMA-2 (vs letrozole):** Neutropenia (82% vs 6%), infections (64% vs 46%), leukopenia (43% vs 3%), arthralgia (41% vs 40%), fatigue (41% vs 29%), nausea (38% vs 27%), alopecia (34% vs 16%), stomatitis (33% vs 15%), diarrhea (30% vs 23%)[1]	**MONALEESA-2 (vs letrozole):** Neutropenia (65% vs 5%), nausea (55% vs 32%), fatigue (43% vs 35%), arthralgia (41% vs 42%), diarrhea (41% vs 26%), alopecia (35% vs 17%), vomiting (35% vs 19%)[3]	**MONARCH-3 (vs AI):** Diarrhea (82% vs 32%), neutropenia (44% vs 2%), fatigue (41% vs 34%), nausea (41% vs 20%), anemia (31% vs 8%), abdominal pain (31% vs 13%), vomiting (30% vs 13%)[7]
	**PALOMA-3 (vs fulvestrant):** Neutropenia (84% vs 3%), leukopenia (60% vs 5%), infections (55% vs 35%), fatigue (44% vs 32%), nausea (36% vs 30%), anemia (32% vs 13%), stomatitis (31% vs 14%)[2]	**MONALEESA-3 (vs fulvestrant):** Neutropenia (74% vs 5%), nausea (45% vs 28%), fatigue (31% vs 33%), leukopenia (33% vs 2%)[4,5]	**MONARCH-2 (vs fulvestrant):** Diarrhea (87% vs 28%), neutropenia (50% vs 4%), nausea (49% vs 25%), fatigue (43% vs 29%), abdominal pain (37% vs 17%), anemia (35% vs 4%), leukopenia (33% vs 2%)[8]

		**MONALEESA-7 (vs ET):** Neutropenia (78% vs 11%), leukopenia (36% vs 6%), infections (57% vs 43%)[6]	
Most common AEs (grade ≥3)[b]	**PALOMA-2 (vs letrozole):** Neutropenia (69% vs 1%), infections (9% vs 3%), leukopenia (27% vs 0%), anemia (7% vs 2%)[1]	**MONALEESA-2 (vs letrozole):** Neutropenia (52% vs 1%), neutrophil count decreased (18% vs 0%), hypertension (16% vs 14%), WBC count decreased (14% vs 1%), increased ALT (11% vs 1%), leukopenia (10% vs 1%), increased AST (6% vs 1%)[3]	**MONARCH-3 (vs AI):** Neutropenia (24% vs 1%), leukopenia (9% vs 1%), diarrhea (9% vs 1%), anemia (7% vs 1%), ALT increased (6% vs 2%)[7]
	**PALOMA-3 (vs fulvestrant):** Neutropenia (70% vs 0%), leukopenia (39% vs 1%), infections (6% vs 3%)[2]	**MONALEESA-3 (vs fulvestrant):** Neutropenia (53% vs 0%), leukopenia (14% vs 0%), increased ALT (7% vs 2%)[5]	**MONARCH-2 (vs fulvestrant):** Neutropenia (30% vs 2%), diarrhea (15% vs 0%), leukopenia (11% vs 0%), anemia (9% vs 1%)[8]
		**MONALEESA-7 (vs ET):** Neutropenia (65% vs 6%), leukopenia (17% vs 2%), hepatobiliary toxicity (11% vs 7%)[6]	
Patients with an AE leading to	**PALOMA-2 (vs letrozole):** 9.7% vs 5.9%[1]	**MONALEESA-2 (vs letrozole):** 7.5% vs 2.1%[10]	**MONARCH-3 (vs AI):** 16.5% vs 3.1%[7]

treatment discontinuation	**PALOMA-3 (vs fulvestrant):** 6.1% vs 4.0%)[2]	**MONALEESA-3 (vs fulvestrant):** 8.5% vs 4.1%[5]	**MONARCH-2 (vs fulvestrant):** 15.9% vs 3.1%[9]
		**MONALEESA-7 (vs ET):** 4.8% vs 3.6%[6]	
**Early breast cancer**			
Most common AEs (all grades)[a]	**PALLAS (vs ET):** Neutropenia (83% vs 5%), leukopenia (55% vs 8%), fatigue (41% vs 19%), arthralgia (38% vs 45%)[11]<br>**PENELOPE-B (vs ET):** Leukopenia (99% vs 70%), neutropenia (96% vs 23%), anemia (74% vs 30%), fatigue (66% vs 51%), infection (60% vs 51%), thrombocytopenia (57% vs 16%), hot flushes (44% vs 51%), arthralgia (41% vs 47%), hypocalcemia (35% vs 24%)[12]		**monarchE (vs ET):** Diarrhea (84% vs 9%), fatigue (41% vs 18%), abdominal pain (36% vs 10%), leukopenia (38% vs 7%), neutropenia (46% vs 6%)[13]
Most common AEs (grade ≥3)[b]	**PALLAS (vs ET):** Neutropenia (62% vs 0%), leukopenia (30% vs 0%)[11]<br>**PENELOPE-B (vs ET):** Neutropenia (70% vs 1%), leukopenia (56% vs 1%)[12]		**monarchE (vs ET):** Diarrhea (8% vs 0%), leukopenia (11% vs 0%), neutropenia (20% vs 1%), lymphopenia (5% vs 1%)[13]
Patients with an AE leading to	**PALLAS (vs ET):** 27.2% vs [14]<br>**PENELOPE-B (vs ET):** 2.6% vs 0.8[12]		**monarchE (vs ET):** 6.4% vs 1.1%[13]

treatment discontinuation

AI = aromatase inhibitor; ALT = alanine aminotransferase; AST = aspartate aminotransferase; ET = endocrine therapy; WBC = white blood cell.

[a]Occurring in >30% of patients in the CDK4/6 inhibitor arm. [b]Occurring in >5% of patients in the CDK4/6 inhibitor arm.

References

1. Finn RS, Rugo HS, Dieras VC, et al. Overall survival (OS) with first-line palbociclib plus letrozole (PAL+LET) versus placebo plus letrozole (PBO+LET) in women with estrogen receptor–positive/human epidermal growth factor receptor 2–negative advanced breast cancer (ER+/HER2− ABC): Analyses from PALOMA-2. *J Clin Oncol.* 2022;40 (LBA 1003).
2. Cristofanilli M, Rugo HS, Im SA, et al. Overall survival with palbociclib and fulvestrant in women with HR+/HER2- ABC: Updated exploratory analyses of PALOMA-3, a double-blind, phase 3 randomized study. *Clin Cancer Res*. 2022;28(16):3433–3442.
3. Hortobagyi GN, Stemmer SM, Burris HA, et al. Overall survival with ribociclib plus letrozole in advanced breast cancer. *N Engl J Med.* 2022;386(10):942–950.

4. Neven P, Fasching PA, Chia S*,* et al*.* LBA4 Updated overall survival (OS) results from the first-line (1L) population in the phase III MONALEESA-3 trial of postmenopausal patients (PTS) with HR+/HER2− advanced breast cancer (ABC) treated with ribociclib (RIB) + fulvestrant (FUL). *Ann Oncol*. 2022;33(suppl 3):S194–S223.

5. Slamon DJ, Neven P, Chia S*,* et al*.* Phase III randomized study of ribociclib and fulvestrant in hormone receptor-positive, human epidermal growth factor receptor 2-negative advanced breast cancer: MONALEESA-3. *J Clin Oncol.* 2018;36(24):2465–2472.

6. Lu YS, Im SA, Colleoni M*,* et al*.* Updated overall survival of ribociclib plus endocrine therapy versus endocrine therapy alone in pre- and perimenopausal patients with HR+/HER2- advanced breast cancer in MONALEESA-7: A phase III randomized clinical trial. *Clin Cancer Res*. 2022;28(5):851–859.

7. Johnston S, Martin M, Di Leo A*,* et al*.* MONARCH 3 final PFS: a randomized study of abemaciclib as initial therapy for advanced breast cancer. *NPJ Breast Cancer*. 2019;5:5.

8. Sledge GW, Jr., Toi M, Neven P*,* et al*.* The effect of abemaciclib plus fulvestrant on overall survival in hormone receptor-positive, ERBB2-negative breast cancer that progressed on endocrine therapy-MONARCH 2: A randomized clinical trial. *JAMA Oncol*. 2020;6(1):116–124.

9. Sledge GW, Jr., Toi M, Neven P*,* et al*.* MONARCH 2: Abemaciclib in combination with fulvestrant in women with HR+/HER2- advanced breast cancer who had progressed while receiving endocrine therapy. *J Clin Oncol.* 2017;35(25):2875–2884.

10. Hortobagyi GN, Stemmer SM, Burris HA, et al. Updated results from MONALEESA-2, a phase III trial of first-line ribociclib plus letrozole versus placebo plus letrozole in hormone receptor-positive, HER2-negative advanced breast cancer. *Ann Oncol*. 2018;29(7):1541–1547.

11. Gnant M, Dueck AC, Frantal S, et al. Adjuvant palbociclib for early breast cancer: the PALLAS trial results (ABCSG-42/AFT-05/BIG-14-03). *J Clin Oncol.* 2022;40(3):282–293.

12. Loibl S, Marme F, Martin M, et al. Palbociclib for residual high-risk invasive HR-positive and HER2-negative early breast cancer – The Penelope-B trial. *J Clin Oncol.* 2021;39(14):1518–1530.

13. Johnston SRD, Toi M, O’Shaughnessy J, et al. Abemaciclib plus endocrine therapy for hormone receptor-positive, HER2-negative, node-positive, high-risk early breast cancer (monarchE): results from a preplanned interim analysis of a randomised, open-label, phase 3 trial. *Lancet Oncol*. 2023;24:77–90.

14. Mayer EL, Dueck AC, Martin M, et al. Palbociclib with adjuvant endocrine therapy in early breast cancer (PALLAS): interim analysis of a multicentre, open-label, randomised, phase 3 study. *Lancet Oncol*. 2021;22(2):212–222.

Supplementary Figure 1. Overview of phase III trials of CDK4/6 inhibitors in early and advanced breast cancer. AI = aromatase inhibitor; DDFS = distant disease-free survival; DRFS = distant relapse-free survival; ET = endocrine therapy; IDFS = invasive disease-free survival; L = line; OS = overall survival; PFS = progression-free survival.

*Interim OS results.

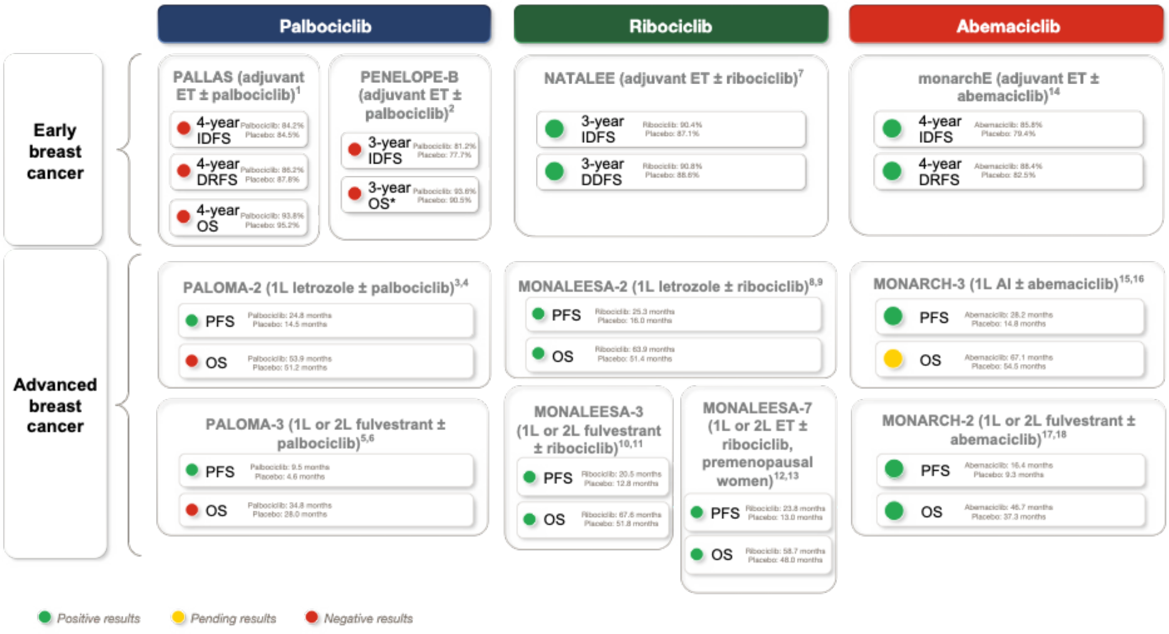

References

1. Gnant M, Dueck AC, Frantal S, et al. Adjuvant palbociclib for early breast cancer: the PALLAS trial results (ABCSG-42/AFT-05/BIG-14-03). *J Clin Oncol.* 2022;40(3):282–293.
2. Loibl S, Marme F, Martin M, et al. Palbociclib for residual high-risk invasive HR-positive and HER2-negative early breast cancer – The Penelope-B trial. *J Clin Oncol.* 2021;39(14):1518–1530.
3. Finn RS, Martin M, Rugo HS, et al. Palbociclib and letrozole in advanced breast cancer. N Engl J Med 2016;375(20):1925–1936.
4. Finn RS, Rugo HS, Dieras VC, et al. Overall survival (OS) with first-line palbociclib plus letrozole (PAL+LET) versus placebo plus letrozole (PBO+LET) in women with estrogen receptor–positive/human epidermal growth factor receptor 2–negative advanced breast cancer (ER+/HER2− ABC): Analyses from PALOMA-2. *J Clin Oncol.* 2022;40 (LBA 1003).

5. Cristofanilli M, Turner NC, Bondarenko I, et al. Fulvestrant plus palbociclib versus fulvestrant plus placebo for treatment of hormone-receptor-positive, HER2-negative metastatic breast cancer that progressed on previous endocrine therapy (PALOMA-3): final analysis of the multicentre, double-blind, phase 3 randomised controlled trial. *Lancet Oncol*. 2016;17(4):425–439.
6. Cristofanilli M, Rugo HS, Im SA, et al. Overall survival with palbociclib and fulvestrant in women with HR+/HER2- ABC: Updated exploratory analyses of PALOMA-3, a double-blind, phase 3 randomized study. *Clin Cancer Res*. 2022;28(16):3433–3442.
7. Slamon DJ, Stroyakovskiy D, Yardley DA, et al. Ribociclib and endocrine therapy as adjuvant treatment in patients with HR+/HER2− early breast cancer: Primary results from the phase III NATALEE trial. *J Clin Oncol.* 2023;41(Suppl 17):LBA500.
8. Hortobagyi GN, Stemmer SM, Burris HA, et al. Updated results from MONALEESA-2, a phase III trial of first-line ribociclib plus letrozole versus placebo plus letrozole in hormone receptor-positive, HER2-negative advanced breast cancer. *Ann Oncol*. 2018;29(7):1541–1547.
9. Hortobagyi GN, Stemmer SM, Burris HA, et al. Overall survival with ribociclib plus letrozole in advanced breast cancer. *N Engl J Med.* 2022;386(10):942–950.
10. Slamon DJ, Neven P, Chia S, et al. Phase III randomized study of ribociclib and fulvestrant in hormone receptor-positive, human epidermal growth factor receptor 2-negative advanced breast cancer: MONALEESA-3. *J Clin Oncol.* 2018;36(24):2465–2472.
11. Neven P, Fasching PA, Chia S, et al. LBA4 Updated overall survival (OS) results from the first-line (1L) population in the phase III MONALEESA-3 trial of postmenopausal patients (PTS) with HR+/HER2− advanced breast cancer (ABC) treated with ribociclib (RIB) + fulvestrant (FUL). *Ann Oncol*. 2022;33(suppl 3):S194–S223.
12. Tripathy D, Im SA, Colleoni M, et al. Ribociclib plus endocrine therapy for premenopausal women with hormone-receptor-positive, advanced breast cancer (MONALEESA-7): a randomised phase 3 trial. *Lancet Oncol*. 2018;19(7):904–915.
13. Lu YS, Im SA, Colleoni M, et al. Updated overall survival of ribociclib plus endocrine therapy versus endocrine therapy alone in pre- and perimenopausal patients with HR+/HER2- advanced breast cancer in MONALEESA-7: A phase III randomized clinical trial. *Clin Cancer Res*. 2022;28(5):851–859.
14. Johnston SRD, Toi M, O'Shaughnessy J, et al. Abemaciclib plus endocrine therapy for hormone receptor-positive, HER2-negative, node-positive, high-risk early breast cancer (monarchE): results from a preplanned interim analysis of a randomised, open-label, phase 3 trial. *Lancet Oncol*. 2023;24:77–90.
15. Johnston S, Martin M, Di Leo A, et al. MONARCH 3 final PFS: a randomized study of abemaciclib as initial therapy for advanced breast cancer. *NPJ Breast Cancer*. 2019;5:5.
16. Eli Lilly. *Verzenios Summary of Product Characteristics*. https://www.ema.europa.eu/en/documents/product-information/verzenios-epar-product-information_en.pdf.

17. Sledge GW, Jr., Toi M, Neven P, et al. MONARCH 2: Abemaciclib in combination with fulvestrant in women with HR+/HER2- advanced breast cancer who had progressed while receiving endocrine therapy. *J Clin Oncol.* 2017;35(25):2875–2884.
18. Sledge GW, Jr., Toi M, Neven P, et al. The effect of abemaciclib plus fulvestrant on overall survival in hormone receptor-positive, ERBB2-negative breast cancer that progressed on endocrine therapy-MONARCH 2: A randomized clinical trial. *JAMA Oncol*. 2020;6(1):116–124.

Supplementary Figure 2. IDFS according to treatment arm and Ki-67 level in Cohort 1 of the monarchE trial, showing prognostic but not predictive effect of Ki-67. (Adapted from Johnston et al. [1]) CI = confidence interval; ET = endocrine therapy; HR = hazard ratio; IDFS = invasive disease-free survival.

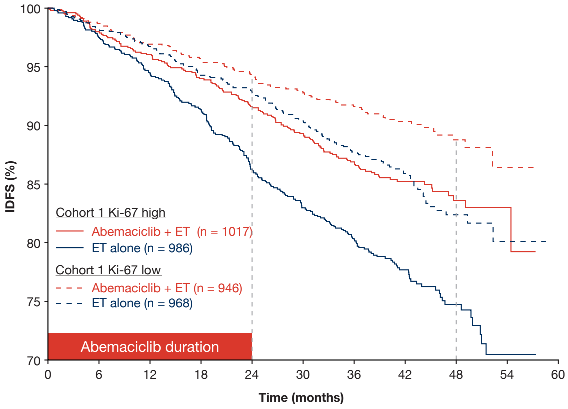

	Abemaciclib + ET	ET alone	HR (95% CI)
**Cohort 1 Ki-67 high, n = 2003**			
No. of events/ No. of patients (%)	147/1017 (14%)	224/986 (23%)	0.62 (0.50–0.76)
4-year IDFS	83.6%	74.7%	
**Cohort 1 Ki-67 low, n = 1914**			
No. of events/ No. of patients (%)	91/946 (10%)	141/968 (15%)	0.62 (0.48–0.81)
4-year IDFS	88.8%	82.4%	
		Ki-67 is prognostic	Ki-67 is not predictive of abemaciclib benefit

Reference

1. Johnston S, Toi M, O’Shaughnessy J, et al. Abemaciclib plus endocrine therapy for HR+, HER2-, node-positive, high-risk early breast cancer: results from a pre-planned monarchE overall survival interim analysis, including 4-year efficacy outcomes. *Cancer Res*. 2023;83(5 suppl):GS1–09.
